# Antimicrobial resistance among migrants in Europe: a systematic review and meta-analysis – update from 2017 to 2023

**DOI:** 10.1016/j.eclinm.2024.102801

**Published:** 2024-09-05

**Authors:** Bridget Chukwudile, Daniel Pan, Luisa Silva, Mayuri Gogoi, Amani Al-Oraibi, Paul Bird, Nisha George, Hayley A. Thompson, Rebecca F. Baggaley, Sally Hargreaves, Manish Pareek, Laura B. Nellums

**Affiliations:** a Nottingham Centre for Public Health and Epidemiology, Lifespan and Population Health, School of Medicine, University of Nottingham, Nottingham, United Kingdom; b Development Centre for Population Health, University of Leicester, Leicester, United Kingdom; c Department of Respiratory Sciences, University of Leicester, Leicester, United Kingdom; d Leicester NIHR Biomedical Research Centre, Leicester, United Kingdom; e Department of Infectious Diseases and HIV Medicine, University Hospitals of Leicester, Leicester, NHS Trust, United Kingdom; f Li Ka Shing Centre for Health Information and Discovery, Oxford Big Data Institute, Oxford, University of Oxford, United Kingdom; g WHO Collaborating Centre for Infectious Disease Epidemiology and Control, School of Public Health, Li Ka Sing Faculty of Medicine, University of Hong Kong, Hong Kong, China; h Department of Microbiology, University Hospitals of Leicester NHS Trust, Leicester, United Kingdom; i Department of Medicine, Imperial College London, London, United Kingdom; j Global Health Programs Division, PATH, Seattle, WA, USA; k Department of Population Health Sciences, University of Leicester, Leicester, United Kingdom; l Migrant Health Research Group, Institute for Infection and Immunity, St George's, University of London, London, United Kingdom; m NIHR Applied Research Collaboration East Midlands, Leicester, United Kingdom; n College of Population Health, University of New Mexico, Albuquerque, NM, United States

**Keywords:** Antimicrobial resistance, Bacteria, Migrants, Refugees, Europe/EU-15 or EEA

## Abstract

**Background:**

Antimicrobial resistance (AMR) is a critical global health concern. A previous systematic review showed that migrants in Europe are at increased risk of AMR. Since the COVID-19 pandemic there have been rapid changes in patterns of antibiotic use, AMR, and migration. We aimed to present an updated evidence synthesis on the current distribution of AMR among migrants in Europe.

**Methods:**

We carried out a systematic review and meta-analysis in accordance with PRISMA guidelines (PROSPERO ID: CRD42022343263). We searched databases (MEDLINE, Embase, PubMed and Scopus) from 18 January 2017 until 18 January 2023 to identify primary data from observational studies reporting any laboratory-confirmed AMR among migrants in the European Economic Area (EEA) and European Union-15 (EU-15) countries using over 7 key search terms for migrants and over 70 terms for AMR and countries in Europe. Outcomes were infection with, or colonisation of AMR bacteria. Methodological quality was assessed using Joanna Briggs Institute Critical Appraisal Checklist for Observational Studies. We meta-analysed the pooled-prevalence of infection and/or colonisation of AMR organisms.

**Findings:**

Among 630 articles, 21 observational studies met the inclusion criteria and were included in this review. The pooled prevalence for any detected AMR was 28.0% (95% CI 18.0%–41.0%, *I*^*2*^ = 100%) compared to a 25.4% seen in the previous review; gram-negative bacteria 31.0% (95% CI 20.0%–44.0%, *I*^*2*^ = 100%), and methicillin-resistant staphylococcus aureus 10.0% (95% CI 5.0%–16.0%, *I*^*2*^ = 99%). Drug-resistant bacteria were more prevalent in community settings in large migrant populations (pooled prevalence: 41.0%, 95% CI 24.0%–60.0%, *I*^*2*^ = 99%) than in hospitals (21.0%, 95% CI 12.0%–32.0%, *I*^*2*^ = 99%). AMR estimates in ‘other’ migrants were 32.0%, (95% CI 12.0%–57.0%, *I*^*2*^ = 100%) and 28.0% (95% CI 18.0%–38.0%, *I*^2^ = 100%) in forced migrants. No firm evidence of AMR acquisition with arrival time or length of stay in the host country was found.

**Interpretation:**

Studies investigating AMR in migrants are highly heterogenous. However, since the COVID-19 pandemic, migrants may be at higher risk of acquiring resistant bacteria, particularly gram-negative bacteria, within community settings such as refugee camps and detention centres in Europe. Our study highlights the importance of infrastructure and hygiene measures within these settings, to mitigate transmission of resistant pathogens. Policy-makers should screen for AMR in migrants prior to departure from countries of origin, where feasible, and upon arrival to a new country to ensure optimal health screening, infection control and effective treatment.

**Funding:**

There was no funding source for this study.

**Research in context**

**Evidence before this study**

Antimicrobial resistance (AMR) is a global concern, especially within migrants. Previous work has shown that COVID-19 may have accelerated AMR, particularly for gram-negative organisms. Prior to the COVID-19 pandemic, a previous systematic review found the prevalence of AMR in Europe to be 25.4% within migrants. Given the changing patterns of migration and the influence of antibiotic use following the COVID-19 pandemic, there was a need for an update regarding the distribution of AMR among migrants to Europe.

**Added value of this study**

This systematic review and meta-analysis was conducted to identify and synthesise data on AMR, including colonisation or infection, in migrants to countries in Europe and the EU/EEA up to January 2023. The pooled prevalence for any detected AMR was 28.0% (95% CI 18.0%–41.0%, *I*^*2*^ = 100%) compared to a 25.4% seen in the previous review. Our findings show high rates of any AMR colonisation or infection among ‘other’ migrants and refugees and asylum seekers, and elevated rates in community settings compared to hospitals. We note a particularly high prevalence of gram-negative drug-resistant organisms amongst migrants in Europe, which may reflect the types of congregant settings in which these organisms are transmitted.

**Implications of all the available evidence**

We show that within Europe, the prevalence of AMR in migrants, particularly within refugees and asylum seekers is increasing; particularly in community settings. These will often be refugee camps, transit hubs or detention facilities within receiving countries. Our results demonstrate the vulnerability of migrant communities to AMR exposure in Europe and the urgent need for interventions to better prevent, detect, and treat AMR infections in these settings, in line with better social, environmental and health conditions.

## Introduction

Antibiotics treat and prevent common infections in humans and animals.^1^ Extensive use of antibiotics use contribute to antimicrobial resistance (AMR).^2^ The most common bacteria linked to mortality from AMR are *Streptococcus pneumoniae, Acinetobacter baumannii, Staphylococcus aureus, Pseudomonas aeruginosa, Escherichia coli*, and *Klebsiella pneumoniae*.^3^^,^^4^ Often, mortality from AMR is exacerbated within settings caused by overcrowding, or poor water and sanitary conditions, which in turn often occurs in human migration.^5^

Addressing AMR is becoming increasingly challenging. Between 2001 and 2014, resistance to third-generation cephalosporins in gram-negative bacteria increased by 13.3% in Europe.^6^ In England, E.coli resistance to piperacillin-tazobactam increased from 8·5% to 11·7%, while the resistance level of *K. pneumoniae* increased by 5.9% between 2011 and 2015.^7^ Meanwhile, in 2015, 63.5% of the 671,689 infections caused by AMR in the EU/EEA were linked to healthcare settings.^8^

Over 87 million migrants are residents in Europe, with 37.5% born outside the EU.^9^ While migrants constitute a diverse community, some may be at increased risk of AMR due to several factors, including exposure to illnesses, limited or interrupted access to healthcare which COVID-19 may have worsened, and unsuitable living circumstances before, during, and after arrival in receiving countries.^10^, ^11^, ^12^ Furthermore, during the pandemic, antibiotics were frequently prescribed for patients with COVID-19, despite absence of evidence of a superadded bacterial infection.^8^ We therefore performed a systematic review and meta-analysis to investigate trends of AMR amongst migrants in Europe, following a period of mass changes in global antmicrobial prescribing following the COVID-19 pandemic. Our findings have public health implications for understanding the burden of AMR amongst migrants in Europe.

## Methods

This systematic review and meta-analysis followed the Preferred Reporting Items for Systematic Reviews and Meta-Analyses guidelines (PRISMA),^13^ and the review protocol was registered with PROSPERO (CRD42022343263).

### Data sources and searches

Search strategies and search terms were developed from similar research and previous systematic reviews in migrant health and AMR.^2^^,^^12^ We searched Embase, MEDLINE, PubMed, and Scopus for articles reporting primary findings from observational studies between January 18, 2017 and January 18, 2023. This start date was chosen since it is a follow-up study from a previous systematic review on AMR in migrants to Europe, reporting evidence up until January 18, 2017.^12^ A Boolean search strategy with search terms relating to migration, AMR, bacterial infections, EU-15 and EEA countries, and the appropriate MeSH headings was used for each database. Appendix I details the specific database search strategies and the number of studies found. Migrants were classified as persons born outside the country where the study was conducted, including forced migrants (e.g asylum seekers, refugees, migrant children) and ‘other’ migrant groups. Forced migrants were categorised as persons subjected to leaving their country of residence due to threats to life and livelihood, such as environmental disasters, political unrest, war, persecution, and famine.^14^ ‘Other’ migrants were foreign-born and had migrated for different reasons, including work, education or reuniting with family.

Studies that examined drug resistance in tuberculosis were excluded.^12^ We also excluded articles in which migrant status was not defined or was determined by ethnicity, country of birth of participants' parents, and articles in which data were not separated or reported by migrant status. Studies that did not present original data or reported non-laboratory confirmed data on AMR, including editorials, comments, reviews, letters, and case reports, were also excluded. No language restrictions were placed on the searches or search results.

### Outcomes

Our primary outcome was infection, or colonisation with laboratory confirmed antibiotic resistant organisms, such as methicillin-resistant *Staphylococcus aureus* (MRSA) and gram-negative bacteria, including extended-spectrum β-lactamase producing Enterobacteriaceae (ESBL-PE) and multidrug-resistant bacteria (combined resistance to three or more class of antibiotics).

### Study selection and data collection

BC and LBN screened the bibliographies of included articles to identify additional eligible studies. Title and abstract screening, full-text screening, data extraction, and quality assessment were done independently. Any discrepancies were discussed until a mutual agreement was reached.

### Data extraction and quality assessment

Mendeley V1.19.8 and Rayyan software were used to manage references, deduplication, and for screening. Data were extracted using a predesigned excel sheet and based on study design, study setting, type of migrant, country of study, and outcome reported. Methodological quality assessment of articles was done using the Joanna Briggs Institute Critical Appraisal (JBI) tools for observational studies.^15^ The tool consisted of an 11-point scale for cohort studies and an 8-point scale for cross-sectional studies evaluating descriptions of the study population and setting, inclusion criteria, accounting for confounders and use of appropriate statistical methods. Articles were given a quality score percentage to reflect methodological rigour and clarity in reporting. Articles were not excluded based on their quality scores, although we did conduct sensitivity analysis to ascertain the robustness of our findings.

### Statistics

Eligible studies that reported AMR prevalence were included in the meta-analysis. Data analysis was done in R V4.1.1 using the *meta-packages* to estimate the pooled AMR prevalence and 95% confidence intervals. Random-effects models were used to account for heterogeneity in the study, which was assessed through the *I*^2^ statistic.DerSimonian and Laird estimator and Freeman-Tukey double arcsine transformations were used to account for variations in the true effect between and within studies.^16^ For all migrants, pooled estimates of the prevalence of AMR colonisation and infection were calculated, and stratified based on migrant type and settings. Sub-analyses were also performed for MRSA and drug-resistant gram-negative bacteria. Heterogeneity was graphically explored in forest plots to check potential sources which could be explained by study setting, migrant type, screening approaches and sample processing. Funnel plots and Eggers’ test were used to check for asymmetry between included studies.

### Role of funding source

No funding for this study.

### Ethics

No ethical approval was required for this study as we are compiling existing, published data.

## Results

### Study selection

**Fig. 1:**
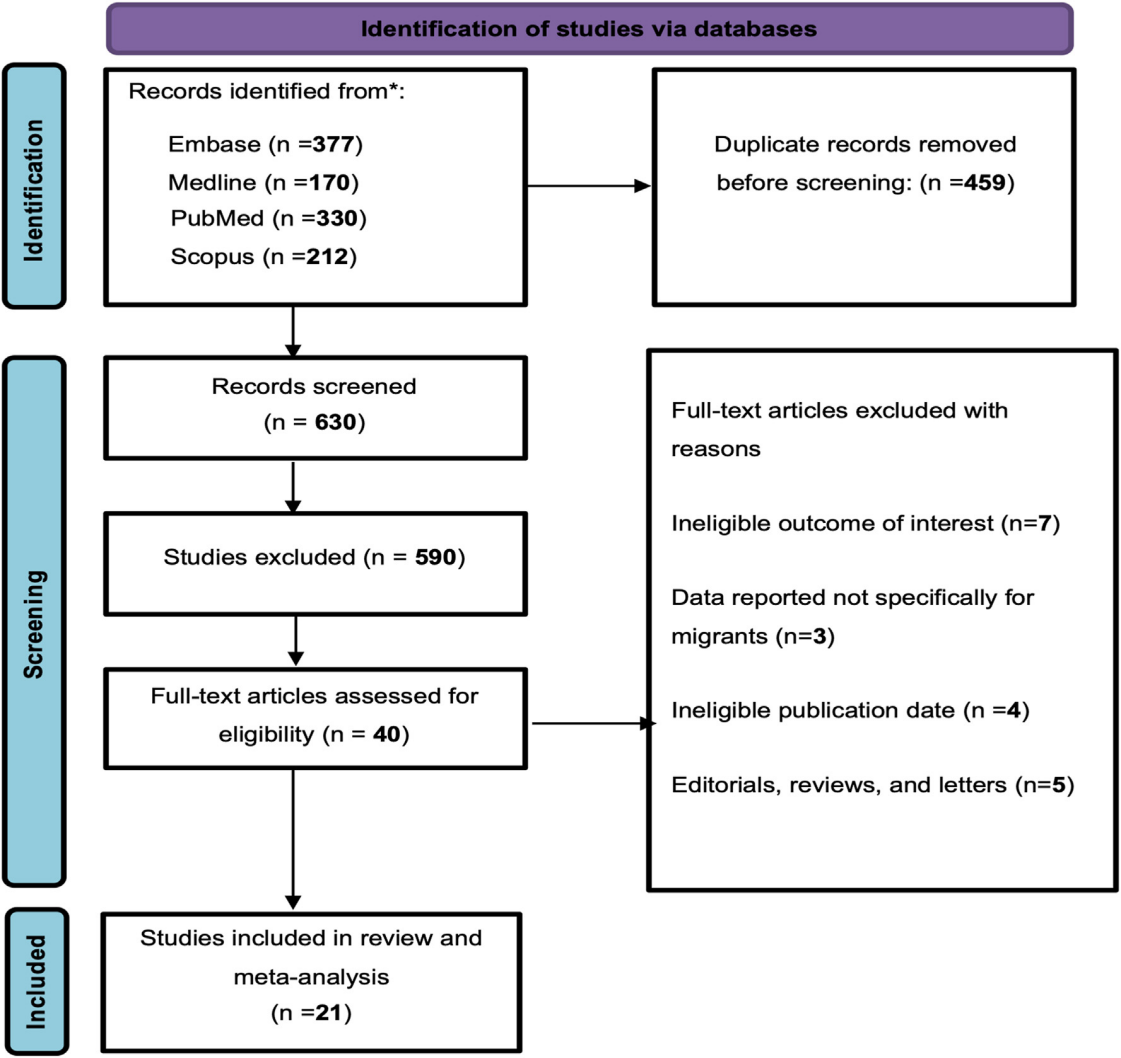
PRISMA flow diagram, illustrating the flow of studies from identification to inclusion.

1089 articles were identified in the database search of published literature, as shown in Fig. 1. After removing 459 duplicates, 630 articles were assessed for eligibility, of which 40 were included for full-text screening. Of the 40 articles included for full-text screening, 19 articles did not meet the inclusion criteria (see Fig. 1). The final 21 studies^17^, ^18^, ^19^, ^20^, ^21^, ^22^, ^23^, ^24^, ^25^, ^26^, ^27^, ^28^, ^29^, ^30^, ^31^, ^32^, ^33^, ^34^, ^35^, ^36^, ^37^ that met inclusion criteria and reported data on AMR either as colonisation or infections in 14,168 migrants were included in this meta-analysis.

### Descriptive characteristics of included studies

**Fig. 2:**
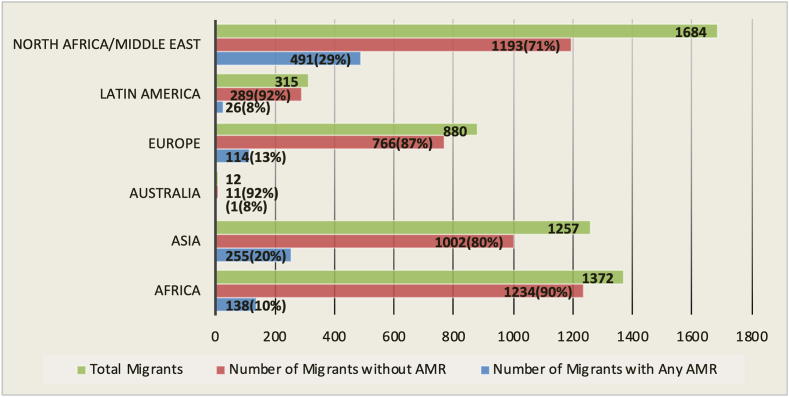
Distribution of AMR organisms according to migrants region of origin. Abbreviations used: AMR: antimicrobial resistance.

Of the 21 studies included, six were conducted in Germany,^19^^,^^20^^,^^28^^,^^29^^,^^32^^,^^34^ two in France,^26^^,^^36^ Italy,^18^^,^^22^ Denmark,^21^^,^^35^ and The Netherlands,^31^^,^^37^ and two in the European Union (a combination of samples from Austria, France, Finland, Germany, Netherland, Spain, Switzerland).^30^^,^^33^ In addition, one was done each in Finland,^17^ Greece,^27^ Sweden,^24^ Spain,^23^ and Switzerland.^25^ Out of the 14,168 migrants included in this review, 6009 (42.4%) were forced migrants (e.g refugees or asylum seekers),^17^^,^^19^, ^20^, ^21^, ^22^^,^^24^, ^25^, ^26^, ^27^, ^28^^,^^31^^,^^32^^,^^34^ and 8159 (57.6%) were migrant children or foreign-born individuals reuniting with their families or migrating for economic or other reasons.^18^^,^^23^^,^^29^^,^^30^^,^^33^^,^^35^, ^36^, ^37^ In six studies, the sample population was children under 18 years,^18^^,^^23^, ^24^, ^25^^,^^27^^,^^29^ while in 13 studies, participants were adults aged 18–82 years.^17^^,^^19^^,^^21^^,^^22^^,^^26^^,^^28^^,^^30^^,^^32^, ^33^, ^34^, ^35^, ^36^, ^37^ Two studies did not report the participants' age.^20^^,^^31^ Migrants' regions of origin commonly encountered across the studies were sub-Saharan Africa, Latin America, Asia, Europe and the Middle East, with migrants predominantly coming from Syria, Iraq, Eritrea, Somalia, Pakistan, and Afghanistan (see Fig. 2).

A total of 13 studies occurred in a hospital or clinic,^17^^,^^18^^,^^22^, ^23^, ^24^, ^25^, ^26^^,^^28^, ^29^, ^30^^,^^32^^,^^36^^,^^37^ while eight were conducted in an asylum-seeking or refugee facility, for example, refugee camps or transit or arrival centres.^19^, ^20^, ^21^^,^^27^^,^^31^^,^^33^, ^34^, ^35^ All studies reported prevalence rates of AMR in migrants identified during screening sessions intended for a particular population, such as asylum seekers or refugees, and in specific settings, such as arrival facilities or at the time of hospital admission with an existing infection. In addition, four studies reported time taken to travel to the host country, ranging from 30 days to 350 days, depending on the travel route.^17^^,^^31^^,^^32^^,^^35^

Three studies reported AMR prevalence among refugees based on the length of stay in the host country.^31^^,^^32^^,^^35^ When described, clinical signs of infection were mostly skin and soft tissue infections or diarrhoea. Samples collected for laboratory testing included throat, nasal, and rectal samples, biopsies, wound swabs, and faecal samples. Different guidelines for determining the antibiotic susceptibility of clinical and screening samples used in various investigations and the rules for interpreting the antimicrobial sensitivity and minimum inhibitory concentrations were reported across studies.

All 21 studies recorded colonisation or infection, of one or multiple forms of resistance. Thirteen studies detected MRSA, of which four were community-associated,^19^^,^^21^^,^^32^^,^^35^ ESBL-producing bacteria,^17^^,^^18^^,^^20^^,^^21^^,^^25^^,^^26^^,^^28^^,^^31^^,^^32^^,^^35^^,^^36^ and vancomycin-resistant enterococcus.^17^^,^^29^ Nine studies reported AMR distribution according to the region of origin (see Fig. 2). Using the JBI critical assessment checklist for observational studies, the studies received scores ranging from 60% to 100% on questions about their quality (Appendix II and III). Three studies^17^^,^^21^^,^^35^ accounted for missing data by multiple imputations or by creating a separate group for categorical variables. More than two-thirds of the studies controlled for the effect of at least one covariate (confounder) either by matching or stratifying sample participants or using multivariable regression analysis.^17^, ^18^, ^19^, ^20^, ^21^, ^22^, ^23^^,^^26^^,^^29^^,^^31^, ^32^, ^33^, ^34^, ^35^, ^36^ Furthermore, less than one-third of the included studies reported the travel duration to the host country.^17^^,^^31^^,^^32^^,^^35^ However, none addressed how travel time impacted the development of AMR. Instead, AMR rates were compared with the various durations of stay since migrants' arrival time. A detailed summary of the included studies is shown in Table 1.

**Table 1:** Summary of included studies.

Citation	Study Country	Study design	Study setting	Migrant type	No of migrants.	Study details	Method of analysis	Outcome measure	Quality assessment score
Aro et al. (2018)^17^	Finland	Cohort	Hospital	Forced Migrants	447	Screening of asylum seekers and refugees for MRSA and MDRGN bacteria; samples were collected as swabs from the nostrils, pharynx, rectum and wound infections.	Laboratory culture of swab samples in pre-enrichment media and susceptibility testing. Statistical analyses were conducted with SPPS. Univariate analysis for categorical variables, chi-squared test, or binary logistic regression analysis.	MDR bacteria 45% (201/447), ESBL-PE 32.9% (147/447), MRSA 21.3% (95/447). Carriage rate by region for migrants was Middle East (56%), Asia (38.6%), sub-Saharan Africa (24.4%), and Europe (15.4%).	72%
Costa et al. (2018)^18^	Italy	Cohort	Hospital	Other Migrants	354	Migrant children who underwent cardiac surgery conducted in 2015–2016 were screened upon hospital admission to identify multi-resistant organisms. Nasal and rectal swabs were collected.	MDRO proportions were compared in Italian and non-Italian children with Z-test.	MDRO colonisation rate was significantly different in the non-Italian and Italian groups (61.9% vs 24.8%, P < 0.001). The rate of ESBL-producing Enterobacteriaceae was 60.5%.	81%
Creutz et al. (2022)^19^	Germany	Cross-sectional	Community	Forced Migrants	161	Voluntary screening of refugees living in a communal area for nasal carriage of *S. aureus*. Each participant provided a nasal swab.	Isolates were phenotypically examined for resistance and virulence by PCR and whole genome sequencing.	2.5% colonisation rate with MRSA	100%
Ehlkes et al. (2019)^20^	Germany	Cross-sectional	Community	Forced Migrants	1544	Asylum seekers with a median age of 25 years were sampled for antibiotic-resistant Enterobacteriaceae. Stool samples were collected, and region of origin and demographic features were explored as risk factors for colonisation.	Univariate and multivariable logistic regression modelling to determine potential risk factors for ESBL-PE/C-PE colonisation.	294 migrants tested positive for ESBL-PE colonisation. Asylum seekers from Afghanistan/Pakis/Iran had the highest prevalence of 29.3%, 20.4% from Syria.	100%
Eiset et al. (2020)^21^	Denmark	Cross-sectional	Community	Forced Migrants	113	Adult Syrian asylum seekers newly arriving in Denmark were screened for intestinal parasites and selected antimicrobial-resistant organisms, including Diphtheria, ESBL-PE, MRSA, and CPO. Faecal and throat swabs were collected.	Prevalence of colonisation and antimicrobial resistance were calculated with their corresponding 95% confidence interval.	Antimicrobial resistance was observed in eight individuals, including one ESBL and seven MRSA.	100%
Fiorini et al. (2020)^22^	Italy	Cohort	Hospital	Forced Migrants	294	Diagnosis and treatment of immigrant patients diagnosed with *H. pylori* infection in a single centre with either sequential or pylera therapy.	Means and 95% confidence intervals were derived. Eradication rates were measured by intention-to-treat (ITT) analysis and per-protocol (PP) analysis. Fisher's exact Chi-square test was used to compare treatment groups.	Latin American immigrants had the highest resistance to metronidazole, tetracycline, levofloxacin, and clarithromycin.	63.6%
Garriga et al. (2021)^23^	Spain	Cohort	Hospital	Other Migrants	48	Identification of S.aureus in patients aged 0–16years managed in pediatric emergency departments	Using SPSS, descriptive and inferential statistical analysis were performed to identify potential risk factors associated with morbidity and mortality.	MRSA in children born in Spain was 13.3% versus 52% in those born outside Spain.	81.8%
Hertting et al. (2021)^24^	Sweden	Cross-sectional	Hospital	Forced Migrants	160	Antimicrobial screening and identification of reasons for hospitalisation in asylum-seeking children less than 18 years.	A severity measure was based on the number of events leading to acute care admission, screening records of MRSA and ESBL-pe colonisation rate among asylum-seeking children/adolescents and compared with the resident population. A Chi-square test was used.	The colonisation rate for MRSA and ESBL-PE was 12% (27) and 17% (19), respectively.	87.5%
Kenfak-Foguena et al. (2021)^25^	Switzerland	Cross-sectional	Hospital	Forced Migrants	59	Screening of asylum seekers in two different hospitals. Nasal, rectal and throat swabs were collected.	Identification and incubation of bacteria cells with whole genome sequencing.	No association between colonisation with MDR bacteria and with hospitalisation or recent (<3 months) arrival in Switzerland (P = 0.41)	62.5%
Lemoine et al. (2022)^26^	France	Cohort	Hospital	Forced Migrants	139	Unaccompanied refugee minors <18 years arriving in Angiers, western France were screened for intestinal and multi-drug resistant bacteria after arrival. Rectal swabs were collected.	Colonisation rates of bacterial isolates with molecular typing.	Only two bacteria species were identified. Rates of ESBL-PE carriage was 25.7%, and five people were confirmed with *klebsiella pneumonia*	72.7%
Mellou et al. (2021)^27^	Greece	Cross-sectional	Community	Forced Migrants	18	Screening for multidrug-resistant Shigella isolates in a refugee and asylum seeker arrival facility.	Laboratory testing of stool samples using multiplex PCR method.	All eighteen samples were confirmed with three different Shigella species.	87.5%
Kossow et al. (2018)^28^	Germany	Cohort	Hospital	Forced Migrants	225	MRSA and MRGNB screening of refugees admitted to a hospital in Munster.	A Chi-square test was used to compare the prevalence of MRSA in refugee patients and non-refugee patients.	MRSA was seen in 9.8% refugee-patient and MDR-GNB in 12.9%	81.8%
Najeem et al. (2022)^29^	Germany	Cross-sectional	Hospital	Other Migrants	3851	Children admitted to a paediatric hospital were examined for MDRO carriage and risk factors. Swabs were taken from the rectal, throat and nasal areas.	Logistic regression models were used for analysis.	MDRO was 4.31%, MRSA 0.86%, MRGN 3.64%	100%
Nurjadi et al. (2019)^30^	EU	Cross-sectional	Hospital	Other Migrants	374	Surveillance was done in 13 travel clinics admitting patients with travel history and skin and soft tissue infections. Nasal and wound lesion swabs were taken.	Microbiological detection and molecular characterisation of bacterial isolates and regional grouping of MRSA proportions were done with Chi-square.	The prevalence of MRSA was 14%, with the highest proportion in Latin America but low in sub-Saharan Africa.	75%
Ravensbergen et al. (2019)^31^	The Netherland	Cohort	Hospital	Forced Migrant	1789	Retrospective data for asylum seekers registered in the asylum seeker centre were collected from the Certe laboratory system. These include demographic data such as age, sex, sampling date, country of origin and date of arrival in the Netherlands. Throat, rectal and nasal swabs and blood samples were collected.	Samples were screened for MRSA, MDRE, and VRE. Data were analysed with SPSS V.23.0. Descriptive statistics were used for the general characteristics and the duration of MDRO carriage.	MRSA was detected in 185 (9.3%) asylum seekers. 972 asylum seekers were all negative for VRE. 331 (18.5%) asylum seekers were positive for MDRE.	100%
Reinheimer et al. (2019)^32^	Germany	Cohort	Community	Forced Migrants	109	Evaluation of retrospective data collected from refugee patients from refugee accommodation and comparison with non-refugees admitted to the intensive care unit. All patients were screened through nasal and rectal swabs.	Chi-square test	The prevalence for MRSA was 18.3%, ESBL-coli 45.8% and MDRGN 41.3%. According to the length of stay, MDRGN declined from 72.4% (<3 months) to 21.7% (>12 months).	81.8%
Rovirola et al. (2020)^33^	EU	Cohort	Community	Other Migrants	704	Analysis of isolates and patient data reported to the European Gonococcal Surveillance Programme (Euro-GASP) 2010–2014.	Statistical significance was determined by Pearson's χ^2^ test or Fisher's exact test. AMR testing for ceftriaxone, cefixime azithromycin, and ciprofloxacin	AMR isolates in foreign-born patients was 52.0%, n = 366.	72.7%
Saracino et al. (2020)^34^	Germany	Cohort	Community	Other Migrants	103	Biopsies for susceptible tests, culture and histology were collected through endoscopy in migrant patients with *H. pylori* after failure with one treatment.	Means and 95% confidence interval, fishers test and chi-square test to compare treatment groups; eradication rates were calculated.	Resistance was recorded in 57 isolates (55.3%).	63.6%
Sloth et al. (2019)^35^	Denmark	Cohort	Community	Mixed	2824	Urine samples were collected from migrants (refugees and family-reunited migrants) and non-migrants in Denmark.	Stratified analysis was based on migrant status. Odds ratio and antibiotic-resistant patterns were calculated using multivariate logistic regression.	Among migrants, 59.9% of the isolates were Gram- + ve, while 47.2% were Gram -ve.	100%
Stabler et al. (2021)^36^	France	Cross-sectional	Hospital	Other Migrants	101	Estimation of AMR carriage and risk factors in hospitalised and recently arrived migrants	Demographic, migration and living condition and laboratory characteristics were compared between patients with or without any bacteria carriage using Chi-squared r Fishers exact test and the student or Wilcoxon ranked tests for categorical and continuous variables respectively.	Overall resistance was 20.7% including MRSA 5.4% and ESBL 16.3%	87.5%
Van-Dulm et al. (2021)^37^	The Netherland	Cross-sectional	Hospital	Other Migrants	760	Evaluation of HCV, HBV, HIV, and MRSA carriage in undocumented and uninsured migrants (median age 40) through e-swabs.	Demographics and time of exit from the country of origin and arrival to the Netherlands were retrieved. Fisher's test was used for comparison between groups.	Prevalence for MRSA was 2.0%, recorded in 15 participants.	62.5%

∗Abbreviations included: MDRO, multidrug-resistant organisms. MRSA, methicillin-resistant *Staphylococcus aureus*. VRE, vancomycin-resistant-enterococcus. ESBL, extended-spectrum β-lactamase. MRGN, multi-resistant Gram-negative bacteria. HIV, Human Immunodeficiency Virus. HCV, Hepatitis C Virus. HBV, Hepatitis B Virus.

### AMR colonisation

**Fig. 3:**
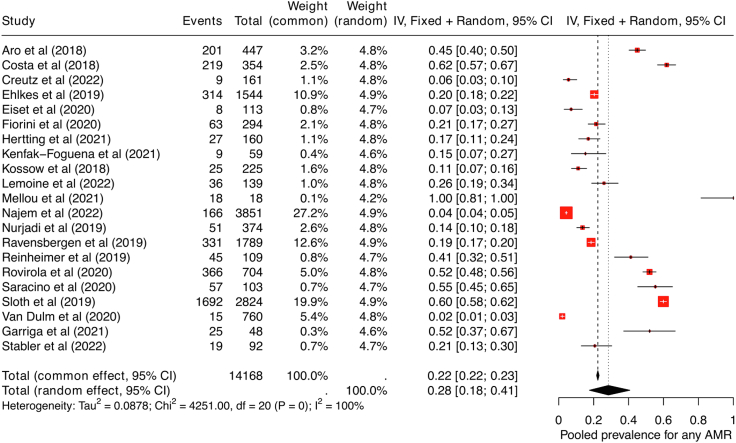
Forest plot showing pooled prevalence of AMR among migrants. Abbreviations used: AMR: antimicrobial resistance.

Overall, the pooled prevalence of AMR colonisation was 28.0% (95% CI 18.0–41.0, *I*^*2*^ = 100%, Fig. 3), with high heterogeneity due to diversity in study populations and settings. The pooled prevalence for colonisation of AMR bacteria across migrants in the included studies was 22·0% (95% CI 10.0–38.0, *I*^*2*^ = 100%), and among those with infection was 41·0% (95% CI 24.0–59.0, *I*^*2*^ = 98%). In addition, an elevated pooled prevalence was seen in drug-resistant gram-negative bacteria (31.0%, 95% CI 20.0–44.0, *I*^*2*^ = 100%) compared to gram-positive bacteria (11.0%, 95% CI 2.0–27.0, *I*^*2*^ = 100%) and MRSA (10.0%, 95% CI 5.0–16.0, *I*^2^ = 99%).

### AMR and settings

**Fig. 4:**
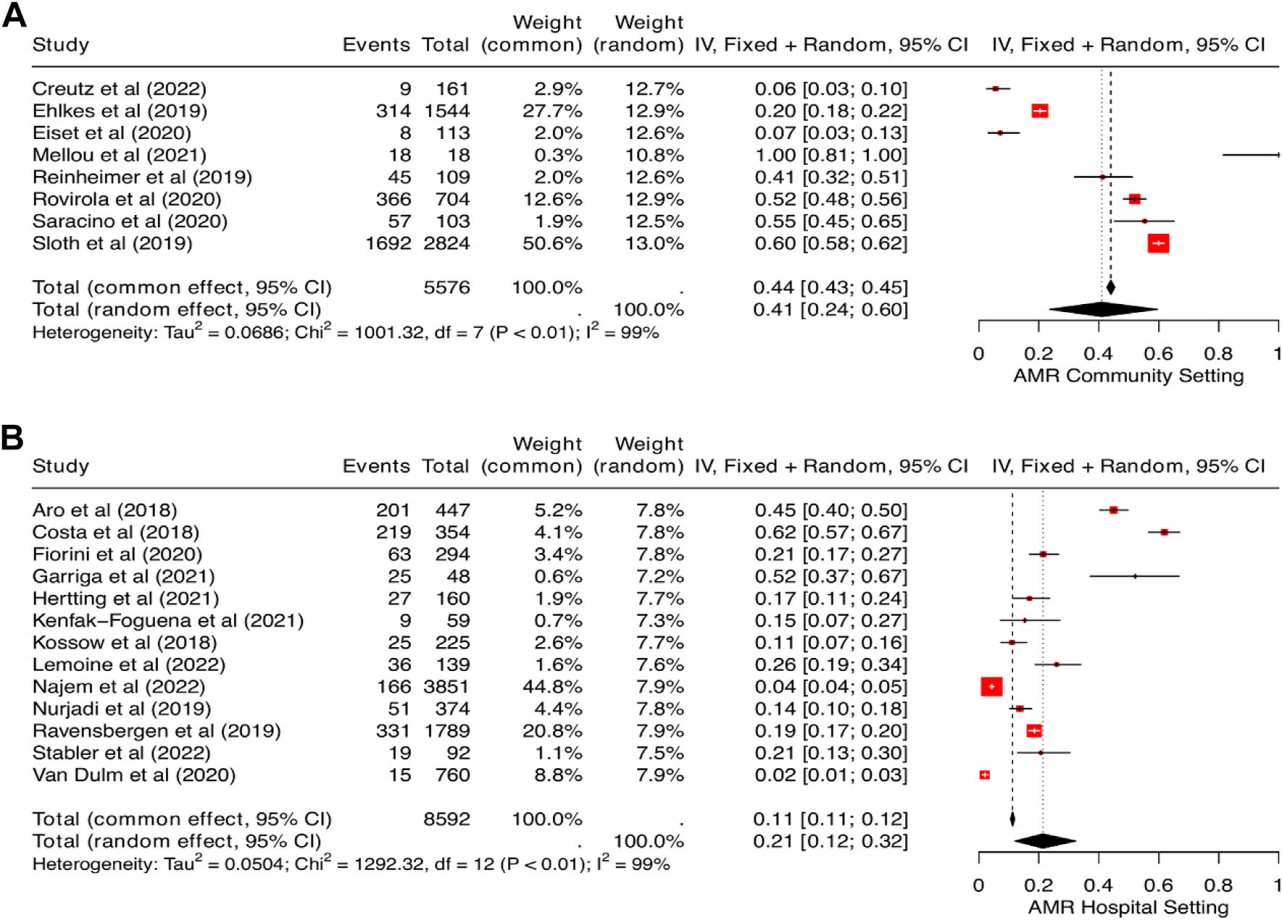
Forest plot showing pooled prevalence of AMR in community (A) and hospital (B) settings. Abbreviations used: AMR: antimicrobial resistance.

In community settings with high numbers of migrants like camps, or transit, and detention centres, the pooled AMR prevalence was 41.0% (95% CI 24.0–60.0, *I*^2^ = 99%, Fig. 4A), and 21.0% pooled AMR prevalence was observed in the hospital settings (95% CI 12.0–32.0, *I*^*2*^ = 99%, Fig. 4B). Our pooled estimates showed more than twofold increases in prevalence of drug-resistant gram-negative bacteria in community setting (52.0%, 95% CI 34.0–69.0, *I*^*2*^ = 99%) compared to hospital settings (23.0%, 95% CI 12.0–37.0, *I*^*2*^ = 99%). Additionally, 11 studies measured the prevalence of MRSA among migrants (as reported in Table 2). For those retrieved from hospital settings, the pooled prevalence of MRSA was 10.0% (95% CI 5.0–16.0 *I*^*2*^ = 99%) and 6.0% in community settings (95% CI 1.0–13.0, *I*^*2*^ = 92%) (Table 3).

**Table 2:** AMR prevalence across migrant settings and migrant groups (Data are pooled prevalence [95% CI]).

	All migrants	Hospital	Community	Other migrants	Forced migrants
Any AMR, carriage, or infection	28.0% (18.0–41.0)	21.0% (12.0–32.0)	41.0% (24.0–60.0)	32.0% (12.0–57.0)	28.0% (18.0–38.0)
Drug-resistant gram-negative	31.0% (20.0–41.0)	23.0% (12.0–35.0)	52.0% (34.0–69.0)	46.0% (37.0–56.0)	27.0% (16.0–39.0)
Methicillin-resistant staphylococcus aureus	10.0% (5.0–16.0)	12.0% (6.0–21.0)	6.0% (1.0–13.0)	9.0% (3.0–20.0)	11.0% (7.0–15.0)

**Table 3:** Actionable strategies to tackle AMR among migrants.

Item	Description
Improved Surveillance/Research	Having reliable and efficient surveillance systems in place is crucial for monitoring the spread and patterns of antimicrobial resistance (AMR) within migrant communities. To achieve this, a unified system must be established to gather data on antimicrobial usage, resistance trends, and healthcare-associated infections. Additionally, it is critical to support research initiatives aimed at uncovering the underlying factors driving AMR among migrant populations, as well as developing innovative methods for prevention, diagnosis, and treatment. This can be achieved through conducting epidemiological studies, genomic surveillance, and clinical trials to inform evidence-based interventions.
Health Education/Stewadrship	Tailored health education campaigns that meet the cultural and linguistic needs of migrant communities are vital for improving their overall health. These campaigns can increase awareness of the appropriate use of antibiotics, the risks of antimicrobial resistance (AMR), and the importance of completing antibiotic courses as prescribed. Additionally, it's crucial to implement antimicrobial stewardship programs in migrant healthcare facilities and provide healthcare providers with training on appropriate prescribing practices. Encouraging the use of diagnostic testing to guide antibiotic treatment decisions is also important. Providing health education and information about AMR, infectious diseases, and appropriate antibiotic use can empower migrants to take control of their health and make informed decisions. Ultimately, this can lead to improved health-seeking behaviors, better adherence to treatment regimens, and reduced reliance on antibiotics.
Restructuring migrant living conditions	Living in overcrowded conditions, such as migrant shelters or poor housing conditions, can cause the rapid spread of infectious diseases, some of which can be resistant to antibiotics. To prevent the transmission of these pathogens, it is crucial to ensure that migrants have access to adequate housing and are not forced to live in overcrowded spaces. Additionally, promoting good hygiene practices, such as hand washing and providing access to clean water and sanitation facilities, can help individuals protect themselves against infections and reduce their dependence on antibiotics. By taking these measures, we can also play a significant role in reducing the selective pressure for antimicrobial resistance.
Access to healthcare	Enhancing the availability of healthcare services for migrant communities is of utmost importance. The language barriers, cultural disparities, and legal status are among the hurdles that they encounter which can impact their physical and mental health. Addressing these obstacles requires culturally knowledgeable healthcare providers, as well as promoting awareness about the existing healthcare resources within migrant communities. Prompt access to healthcare services is equally vital as it aids in the early detection and treatment of infections, thus lessening the likelihood of complications and the use of broad-spectrum antibiotics that contribute to antimicrobial resistance (AMR).
Community Engagement	The involvement of migrant communities is essential to developing and executing effective strategies to prevent antimicrobial resistance (AMR). Accordingly, there is need for robust partnerships with community leaders, advocacy groups, and healthcare providers. In doing so, we can enable these communities to become active participants in their healthcare and efforts to prevent AMR. A supportive environment must be created to facilitate the dissemination of health messages and encourage positive health behaviors, such as the responsible use of antibiotics.

### AMR and migrant type

**Fig. 5:**
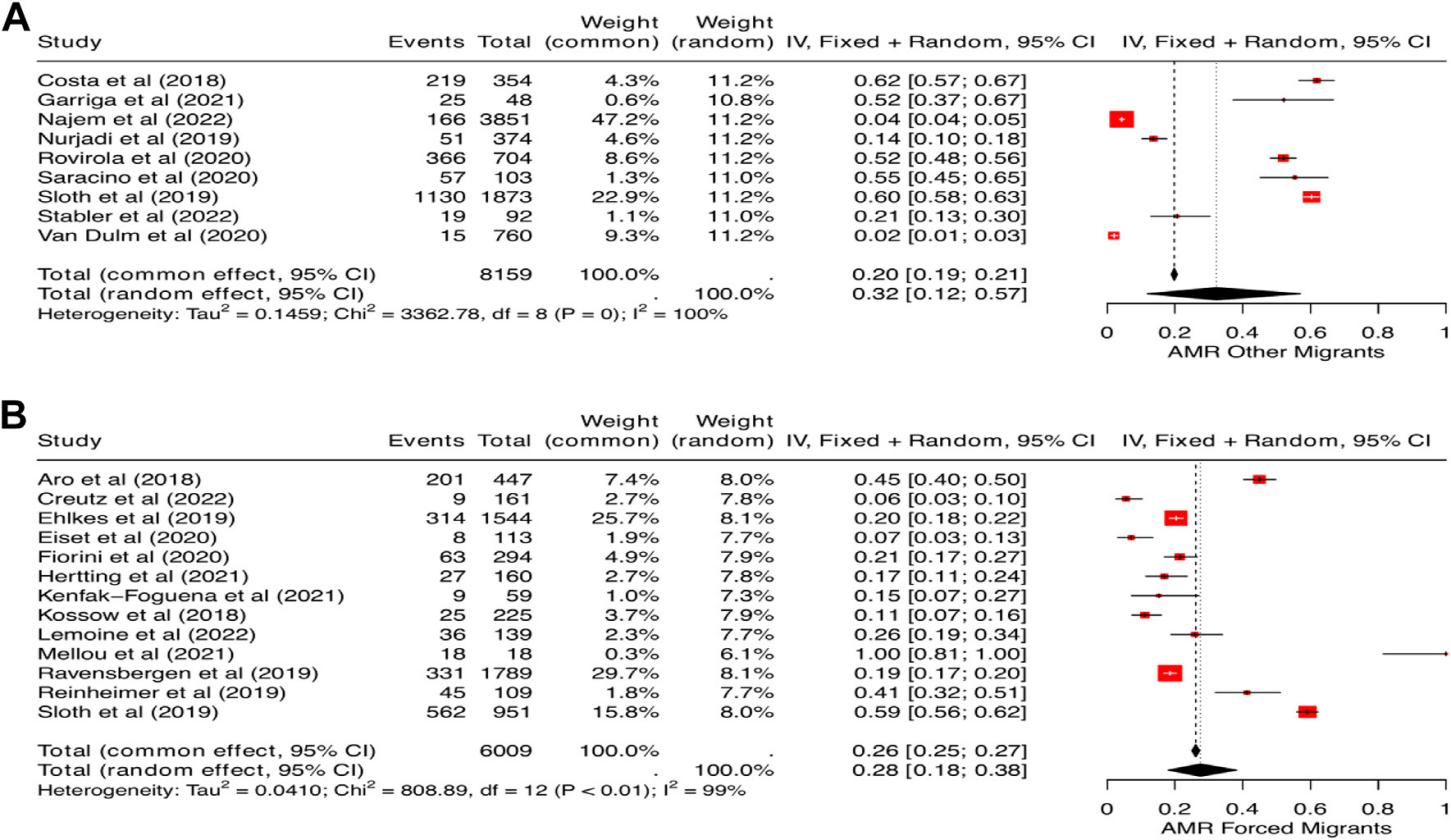
Forest plot showing pooled prevalence of AMR in other migrants (A) and forced migrants (B). Abbreviations used: AMR: antimicrobial resistance.

A total of 14,168 migrants were grouped as either forced migrants or ‘other’ migrants. Overall, 8159 ‘other’ migrants were included across eight studies, while 6009 forced migrants were included across 13 studies. In the pooled prevalence of any identified AMR infection or colonisation among migrant types, the pooled estimate in ‘other’ migrants was 32.0% (95% CI 12.0–57.0, *I*^*2*^ = 100%, Fig. 5A) and in forced migrants: 28.0% (95% CI 18.0–38.0, *I*^*2*^ = 99%, Fig. 5B). Among the 15 studies reporting drug resistance in gram-negative organisms, 11 reported on forced migrants, while four had ‘other’ migrants as participants. Pooled estimates showed a higher prevalence of drug-resistant gram-negative bacteria among ‘other’ migrants (46.0%, 95% CI 37.0–56.0, *I*^*2*^ = 95%) than in forced migrants (27.0%, 95% CI 16.0–39.0, *I*^2^ = 99%). In the subgroup analysis for MRSA prevalence, a higher pooled prevalence was seen in forced migrants (11.0%, 95% CI 7.0–15.0, *I*^*2*^ = 90%) compared to the ‘other’ migrant group (4.0%, 95% CI 0.0–11.0, *I*^*2*^ = 98%). The summary of AMR prevalence across settings and migrant groups is detailed in Table 2.

### AMR and travel time

Three studies investigated colonisation of AMR bacteria with regards to time taken to travel to the receiving country or the length of stay in the receiving country.^31^^,^^32^^,^^35^ Evidence from these studies suggested that the proportion of migrants who tested positive for MRSA or MDRE varied over time. However, there was no discernible trend of reduction or rise.^31^ No evidence was found between AMR acquisition and various migratory routes.

### Sensitivity analysis

Sensitivity analysis, as shown in Appendix IV, was done to assess the influence of article quality on the predicted prevalence of AMR colonisation or infection. Studies with a quality level of 75% or below yielded a pooled prevalence of AMR (26.0%, 95% CI 11.0–46.0, *I*^*2*^ = 99%) that did not vary significantly from when all studies were included (28.0%, 95% CI 18.0–41.0, *I*^*2*^ = 100%). Heterogeneity changes were not significant in subgroups analyses. Funnel plots demonstrated absence of publication bias (Appendix V).

## Discussion

Our study has three main findings. First, we found a high prevalence of AMR colonisation and infection among forced migrants and other migrant groups, particularly in communities with high geographical concentrations of migrants. Second, we found that resistant bacteria were more prevalent in community settings compared to hospitals. Finally, we found that there were low MRSA colonisation rates among migrants, with the majority of AMR attributable to gram-negative multi-drug resistant bacteria.

Our findings showed a higher prevalence of AMR among migrants compared to previous work, suggesting an increase in AMR since the COVID-19 pandemic in Europe.^12^ Many migrants travel from areas where there are minimal antimicrobial stewardship policies compared to the receiving country. Thus, these migrants often come from areas with a high prevalence of AMR organisms in the community, and may bring them to the receiving country. Migration to a receiving country in sub-optimal conditions can also contribute to resistance, depending on route and mode of travel.

When comparing other migrants with forced migrants, a slight difference in the rate of AMR was observed, in contrast to the previous review, but aligns with two studies that found a higher risk of resistance among family-reunited migrants than refugees.^31^^,^^38^ In addition, the prevalence of AMR was observed to differ across regions, with the Middle East/North Africa and Asia having the highest occurrence in migrants. These data needs to be interpreted cautiously, since most studies lacked information about the origin region and corresponding AMR data.

We found a higher prevalence of AMR in migrants within community settings compared to hospitals. Migrants are a highly diverse cohort; with certain subpopulations, such as refugee workers and undocumented migrants facing major difficulties to healthcare services and living within congregant settings, that may increase the risk of both AMR acquisition and transmission. Within healthcare settings, strict infection control procedures and testing limit the spread of AMR. These measures include good hygiene practices, and isolation precautions that are less strict within migrant communities outside the hospital.

Our findings emphasise the role immigrant dominated areas, camps, or transit, arrival, and detention centres might have in increasing the risk of acquiring AMR organisms for migrants. Pathogens which have AMR are more likely to spread in these environments due to poor socio-environmental factors such as overcrowding, improper environmental hygiene, and limited access to adequate health services, including medications or vaccines.^12^^,^^39^^,^^40^ These factors may have a more significant impact in determining AMR among migrants to Europe than the acquisition of resistant bacteria in their countries of origin.^12^ It is possible that a substantial proportion of migrants will have come from refugee routes within recent studies, due to the occurrence of lockdown during the COVID-19 pandemic and ban of travel in many countries within Europe.

Our findings also a greater prevalence of drug-resistant gram-negative bacteria (GNB 31.0%, community 52.0%) than in the prior study (GNB 27.2%, community 32.1%).^12^ The high occurrence of multi-drug resistant gram-negative bacteria could translate to an increased prevalence of urinary tract and gastrointestinal tract infections (GIT) commonly linked with travelling and poor sanitary conditions. Our findings agree with other studies that reported a two to three-fold increase in the colonization of drug-resistant gram-negative bacteria among migrants compared to general community populations in receiving countries.^12^^,^^41^^,^^42^ In addition, a systematic review found that COVID-19 may have accelerated the emergence and transmission of AMR, particularly for gram-negative organisms in hospital settings globally.^43^ Many migrants to Europe travel from countries where high rates of ESBL-PE have been previously reported, such as North Africa and Asia.^42^ Migrants in these areas may be at increased risk of exposure to AMR organisms.^44^

We found that there were low MRSA colonisation rates among migrants, a common cause of skin and soft tissue infections.^45^ However, in migrants who arrive with SSTI, MRSA and Panton-Valentine leucocidin positive (PVL) genes are frequently detected.^30^^,^^46^ The role of MRSA and PVL isolates in spreading AMR genes has been documented in previous research.^45^^,^^47^^,^^48^ However, compared with multidrug-resistant gram-negative bacteria, the risk of MRSA transmission among migrating individuals is substantially lower than gram-negative bacteria.^30^ One reason for why this may be is environmental stability; gram-negative persist longer in the environment due to a robust structural layer that slows down or inhibits the penetration of chemical agents.^49^

It remains unclear whether migrants bring resistant organisms from their country of origin, or whether they acquired the organism in transit, or in refugee centres where living conditions may be limited. Evidence-based data on the prevalence of AMR colonisation in relation to travel time from country of origin or time since arrival in the host country remain limited. One study found a lower *E. coli* resistance (57.6%) in migrants with more than 10 years of stay compared to migrants with less than 5 years of stay (62.6%).^35^ Similarly, in a German study, the prevalence of gram-negative organisms was higher among refugees who recently arrived in Germany (72.4%), with a gradual decline seen after 18 months (14.3%).^32^ This shows that the duration of colonisation with resistant organisms may vary across strains. However, one study found no decrease in the colonisation rate of MDROs among asylum-seekers even after twelve months since arrival.^31^ Meanwhile, studies have shown that resistant organisms may be carried from country of origin to receiving country. For example, blaNDM-1 resistance gene in *P. aeruginosa* was first discovered in North America and Europe, from medical travellers arriving from Asia.^50^ It may be that the spread of resistant bacteria depends on the settings in which a migrant resides, within their host country; if they live in a refugee camp, it may be that they are constantly exposed to resistant pathogens from other refugees and detention centres compared to living with the locals, where they may be a lower prevalence of AMR organisms. More studies involving migrants should aim to record duration since leaving their country of origins to disentangle this issue. Additionally, analysing the genetic makeup of the strains and thoroughly examining their evolutionary relationships could identify information about transmission and clustering.

Our study had limitations. Due to the sampling sites and procedures across the included studies, the colonisation of some resistant organisms may not be detected. Sampling bias, introduced due to requiring a reason for testing (such as treatment failure) could lead to overestimations of AMR prevalence in this study. Notable lack of pre-migration and post-migration tests in many studies reduces the precision and meaningful inferences of the data. Variations in migrant type, settings, bacteria species, type of resistance reported, and standard of measurements utilised differed significantly, resulting in high heterogeneity between studies. Future efforts must focus on strengthening surveillance systems worldwide, ensuring unified reporting, and collecting comprehensive data on migrant patients. Our findings continue to be applicable in 2024; since conducting the study we performed an updated search since the last search date, from January 2023 to March 2024. Our search identified only two additional studies which would not have changed our main findings.^38^^,^^51^

In conclusion, we found an elevated rate of AMR among migrants in Europe since 2017.^12^ The prevalence of AMR in migrants were higher in community settings, especially those with high geographical concentrations of migrants compared to hospitals. The most common causative AMR organisms in migrants were gram-negative bacteria. Our findings emphasise the importance of screening and treating AMR in migrants, especially those from refugee camps. Additionally, policy-makers must engage with migrant communities to ensure that any new health policies are feasible, acceptable and non-stigmatising.

## Contributors

BC, LBN, DP and MP wrote the first draft of the manuscript. BC and LBN collected the data. The remaining authors (LS,MG,AAO,PB,NG,HT,RFB,SH) reviewed and contributed to various revisions of the manuscript and approved the final version for submission. DP and BC have accessed and verified the data. LBN and MP was responsible for the decision to submit the manuscript. All authors read and approved the final version of the manuscript.

## Data sharing statement

All data included were extracted from publicly accessible articles cited in the reference list. Extracted data are presented in this manuscript and appendix.

## Declaration of interests

We declare no competing interests. DP is supported by a NIHR Doctoral Research Fellowship (NIHR302338). The views expressed are those of the authors and not necessarily those of the NIHR or the Department of Health and Social Care. MP is supported by the NIHR Leicester Biomedical Research Centre (BRC) and NIHR Applied Health Collaboration East Midlands, as well as a NIHR Development and Skills Enhancement Award. SH is funded by the NIHR (NIHR300072), the Academy of Medical Sciences (SBF005l1), La Caixa Foundation (LCF/PR/SP21/52930003), Research England, MRC and WHO.
