# Supplementary material for: Antimicrobial resistance among migrants in Europe: a systematic review and meta-analysis – update from 2017 to 2023
Source: eClinicalMedicine. 2024 Sep 5;75:102801. doi: 10.1016/j.eclinm.2024.102801 (PMC11408055; doi:10.1016/j.eclinm.2024.102801)

**Supplementary materials**

**Appendix I. Search strategy**

Database(s): **Ovid MEDLINE(R) ALL**1946 to January 18, 2023.

| **#** | **Searches** | **Results** |
| --- | --- | --- |
| 1 | migrant*.mp. or migrant/ or long distance migrant/ or migrant worker/ | 28042 |
| 2 | population migration/ or migration.mp. or migration/ or migration policy/ or forced migration/ or illegal migration/ | 357980 |
| 3 | immigration/ or immigrant/ or immigra*.mp. | 59755 |
| 4 | refugee camp/ or refugee/ or refugee*.mp. | 17430 |
| 5 | asylum seeker center/ or asylum seeker/ or asylum.mp. | 14882 |
| 6 | foreign born.mp. or foreigner/ | 17624 |
| 7 | foreign-born.mp. or foreigner/ | 17624 |
| 8 | 1 or 2 or 3 or 4 or 5 or 6 or 7 | 430489 |
| 9 | bacterial resistan*.mp. or antibiotic resistance/ or bacterial gene/ | 125497 |
| 10 | antibiotic resistance/ or antibiotic agent/ or antibiotic resistan*.mp. | 107537 |
| 11 | antibiotic resistance/ or bacterial gene/ or antimicrobial resistan*.mp. or antibiotic agent/ | 149330 |
| 12 | extensively drug resistant tuberculosis/ or multidrug resistant tuberculosis/ or XDR.mp. | 11868 |
| 13 | MDR.mp. or multidrug resistant tuberculosis/ or multidrug resistance/ | 45046 |
| 14 | antibiotic resistance/ or multi-drug resistan*.mp. | 72368 |
| 15 | multidrug resistant tuberculosis/ or extensively drug resistant tuberculosis/ or extensively drug-resistan*.mp. or drug resistant tuberculosis/ | 12223 |
| 16 | multidrug resistant tuberculosis/ or extensively drug resistant tuberculosis/ or extensively drug resistan*.mp. or drug resistant tuberculosis/ | 12223 |
| 17 | drug resistance/ or drug resistan*.mp. | 334204 |
| 18 | drug resistance/ or drug-resistan*.mp. | 334204 |
| 19 | antibiotic resistance/ or bacterial gene/ or antimicrobial resistan*.mp. or antibiotic agent/ | 149330 |
| 20 | resistan*.mp. | 1296820 |
| 21 | antibiotic resistance/ or antibiotic sensitivity/ or decreased susceptibility.mp. | 63240 |
| 22 | beta lactamase/ or extended spectrum beta lactamase/ or beta-lactamase*.mp. or ampicillin/ | 53115 |
| 23 | extended spectrum beta lactamase/ or extended- spectrum beta-lactamase*.mp. or beta lactamase/ | 29955 |
| 24 | ESBL.mp. or extended spectrum beta lactamase/ | 9892 |
| 25 | beta lactamase CTX M/ or CTX-M.mp. | 4523 |
| 26 | beta lactamase AmpC/ or AmpC.mp. | 3993 |
| 27 | metallo beta lactamase/ or metallo-beta-lactamase*.mp. | 4378 |
| 28 | MBL.mp. | 7017 |
| 29 | methicillin-resistant Staphylococcus aureus.mp. or methicillin resistant Staphylococcus aureus/ or Staphylococcus aureus/ | 93903 |
| 30 | MRSA.mp. or methicillin resistant Staphylococcus aureus/ | 33376 |
| 31 | 9 or 10 or 11 or 12 or 13 or 14 or 15 or 16 or 17 or 18 or 19 or 20 or 21 or 22 or 23 or 24 or 25 or 26 or 27 or 28 or 29 or 30 | 1418402 |
| 32 | bacteri*.mp. or bacterial infection/ | 1691163 |
| 33 | Escherichia/ or Escherichia coli infection/ or Escherichia coli O157/ or Escherichia coli/ or Escherichia.mp. or Escherichia coli O157:H7/ or multidrug resistant Escherichia coli/ | 419974 |
| 34 | E coli.mp. or Escherichia coli/ | 350056 |
| 35 | Klebsiella/ or Klebsiella pneumoniae infection/ or carbapenem resistant Klebsiella pneumoniae/ or Klebsiella pneumoniae/ or Klebsiella oxytoca/ or Klebsiella*.mp. | 44897 |
| 36 | Staphylococcus infection/ or Staphylococcus.mp. or Staphylococcus/ | 180359 |
| 37 | Streptococcus/ or Streptococcus infection/ or Streptococcus.mp. | 119254 |
| 38 | Salmonella enterica serovar Typhimurium/ or Salmonell*.mp. or Salmonella/ or Salmonella typhimurium/ | 100719 |
| 39 | Shigella flexneri/ or shigellosis/ or Shigell*.mp. or Shigella/ | 22614 |
| 40 | gonorrhea/ or Neisseria gonorrhoeae/ or Gonorrhoeae.mp. | 22654 |
| 41 | gonorrhea.mp. or gonorrhea/ | 17739 |
| 42 | Proteus.mp. or Proteus/ or Proteus infection/ | 19256 |
| 43 | Enterobacter*.mp. or Enterobacter/ | 58832 |
| 44 | Morganella morganii/ or Morganella/ or Morganella.mp. | 1436 |
| 45 | multidrug resistant tuberculosis/ or extensively drug resistant tuberculosis/ or Tuberculosis.mp. or drug resistant tuberculosis/ or tuberculosis/ | 271483 |
| 46 | TB.mp. | 68572 |
| 47 | Yersinia/ or Yersinia*.mp. | 16608 |
| 48 | Gram negative bacterium/ or Gram negative infection/ or Gram negative.mp. | 112273 |
| 49 | ciprofloxacin/ or ofloxacin/ or Fluoroquinolon*.mp. or fluoroquinolone resistance/ | 41306 |
| 50 | Cephalosporin*.mp. or cephalosporin/ or cephalosporin resistance/ | 36602 |
| 51 | carbapenem/ or Carbapenem*.mp. or carbapenem resistance/ | 21578 |
| 52 | Penicillin.mp. or penicillin derivative/ | 70453 |
| 53 | 32 or 33 or 34 or 35 or 36 or 37 or 38 or 39 or 40 or 41 or 42 or 43 or 44 or 45 or 46 or 47 or 48 or 49 or 50 or 51 or 52 | 2307752 |
| 54 | European Union.mp. or European Union/ | 30789 |
| 55 | EU.mp. | 37176 |
| 56 | European Union/ or Europe/ or European Economic Area.mp. | 129602 |
| 57 | European Union/ or EEA.mp. | 19113 |
| 58 | Austria.mp. or Austria/ | 28636 |
| 59 | Belgium.mp. or Belgium/ | 30301 |
| 60 | Britain.mp. or Great Britain/ | 251944 |
| 61 | Czech Republic.mp. or Czech Republic/ | 12916 |
| 62 | Denmark.mp. or Denmark/ | 67218 |
| 63 | England.mp. or United Kingdom/ or England/ | 362058 |
| 64 | Finland.mp. or Finland/ | 49083 |
| 65 | France.mp. or France/ | 139189 |
| 66 | Germany.mp. or Germany/ | 214453 |
| 67 | Greece/ or Greece.mp. | 28151 |
| 68 | Ireland/ or Ireland.mp. | 36630 |
| 69 | Iceland.mp. or Iceland/ | 7375 |
| 70 | Italy.mp. or Italy/ | 141070 |
| 71 | Lichtenstein.mp. | 1447 |
| 72 | Luxembourg.mp. or Luxembourg/ | 1502 |
| 73 | Netherlands/ or Netherlands.mp. | 97948 |
| 74 | Norway.mp. or Norway/ | 59378 |
| 75 | Portugal.mp. or Portugal/ | 22042 |
| 76 | Scotland.mp. or United Kingdom/ or Scotland/ | 274800 |
| 77 | Spain/ or Spain.mp. | 114337 |
| 78 | Sweden.mp. or Sweden/ | 102378 |
| 79 | Switzerland.mp. or Switzerland/ | 53249 |
| 80 | United Kingdom.mp. or United Kingdom/ | 269173 |
| 81 | UK.mp. or United Kingdom/ | 325215 |
| 82 | Wales/ or Wales.mp. | 41282 |
| 83 | 54 or 55 or 56 or 57 or 58 or 59 or 60 or 61 or 62 or 63 or 64 or 65 or 66 or 67 or 68 or 69 or 70 or 71 or 72 or 73 or 74 or 75 or 76 or 77 or 78 or 79 or 80 or 81 or 82 | 1703324 |
| 84 | 8 and 31 and 53 and 83 | 606 |
| 85 | limit 84 to yr="2017 -Current" | 170 |

Database(s): **Embase**1974 to 2023 Week 3

| **#** | **Searches** | **Results** |
| --- | --- | --- |
| 1 | migrant*.mp. or migrant/ or long distance migrant/ or migrant worker/ | 31835 |
| 2 | population migration/ or migration.mp. or migration/ or migration policy/ or forced migration/ or illegal migration/ | 497528 |
| 3 | immigration/ or immigrant/ or immigra*.mp. | 50392 |
| 4 | refugee camp/ or refugee/ or refugee*.mp. | 19063 |
| 5 | asylum seeker center/ or asylum seeker/ or asylum.mp. | 5040 |
| 6 | foreign born.mp. or foreigner/ | 5047 |
| 7 | foreign-born.mp. or foreigner/ | 5047 |
| 8 | 1 or 2 or 3 or 4 or 5 or 6 or 7 | 563885 |
| 9 | bacterial resistan*.mp. or antibiotic resistance/ or bacterial gene/ | 257003 |
| 10 | antibiotic resistance/ or antibiotic agent/ or antibiotic resistan*.mp. | 519448 |
| 11 | antibiotic resistance/ or bacterial gene/ or antimicrobial resistan*.mp. or antibiotic agent/ | 580041 |
| 12 | extensively drug resistant tuberculosis/ or multidrug resistant tuberculosis/ or XDR.mp. | 12798 |
| 13 | MDR.mp. or multidrug resistant tuberculosis/ or multidrug resistance/ | 79868 |
| 14 | antibiotic resistance/ or multi-drug resistan*.mp. | 195421 |
| 15 | multidrug resistant tuberculosis/ or extensively drug resistant tuberculosis/ or extensively drug-resistan*.mp. or drug resistant tuberculosis/ | 14902 |
| 16 | multidrug resistant tuberculosis/ or extensively drug resistant tuberculosis/ or extensively drug resistan*.mp. or drug resistant tuberculosis/ | 14902 |
| 17 | drug resistance/ or drug resistan*.mp. | 317759 |
| 18 | drug resistance/ or drug-resistan*.mp. | 317759 |
| 19 | antibiotic resistance/ or bacterial gene/ or antimicrobial resistan*.mp. or antibiotic agent/ | 580041 |
| 20 | resistan*.mp. | 1739286 |
| 21 | antibiotic resistance/ or antibiotic sensitivity/ or decreased susceptibility.mp. | 245572 |
| 22 | beta lactamase/ or extended spectrum beta lactamase/ or beta-lactamase*.mp. or ampicillin/ | 138769 |
| 23 | extended spectrum beta lactamase/ or extended- spectrum beta-lactamase*.mp. or beta lactamase/ | 38002 |
| 24 | ESBL.mp. or extended spectrum beta lactamase/ | 18280 |
| 25 | beta lactamase CTX M/ or CTX-M.mp. | 5972 |
| 26 | beta lactamase AmpC/ or AmpC.mp. | 5790 |
| 27 | metallo beta lactamase/ or metallo-beta-lactamase*.mp. | 5987 |
| 28 | MBL.mp. | 8154 |
| 29 | methicillin-resistant Staphylococcus aureus.mp. or methicillin resistant Staphylococcus aureus/ or Staphylococcus aureus/ | 203634 |
| 30 | MRSA.mp. or methicillin resistant Staphylococcus aureus/ | 63853 |
| 31 | 9 or 10 or 11 or 12 or 13 or 14 or 15 or 16 or 17 or 18 or 19 or 20 or 21 or 22 or 23 or 24 or 25 or 26 or 27 or 28 or 29 or 30 | 2251775 |
| 32 | bacteri*.mp. or bacterial infection/ | 1723898 |
| 33 | Escherichia/ or Escherichia coli infection/ or Escherichia coli O157/ or Escherichia coli/ or Escherichia.mp. or Escherichia coli O157:H7/ or multidrug resistant Escherichia coli/ | 478521 |
| 34 | E coli.mp. or Escherichia coli/ | 431172 |
| 35 | Klebsiella/ or Klebsiella pneumoniae infection/ or carbapenem resistant Klebsiella pneumoniae/ or Klebsiella pneumoniae/ or Klebsiella oxytoca/ or Klebsiella*.mp. | 84164 |
| 36 | Staphylococcus infection/ or Staphylococcus.mp. or Staphylococcus/ | 289366 |
| 37 | Streptococcus/ or Streptococcus infection/ or Streptococcus.mp. | 177699 |
| 38 | Salmonella enterica serovar Typhimurium/ or Salmonell*.mp. or Salmonella/ or Salmonella typhimurium/ | 112333 |
| 39 | Shigella flexneri/ or shigellosis/ or Shigell*.mp. or Shigella/ | 25041 |
| 40 | gonorrhea/ or Neisseria gonorrhoeae/ or Gonorrhoeae.mp. | 31667 |
| 41 | gonorrhea.mp. or gonorrhea/ | 20719 |
| 42 | Proteus.mp. or Proteus/ or Proteus infection/ | 30757 |
| 43 | Enterobacter*.mp. or Enterobacter/ | 84567 |
| 44 | Morganella morganii/ or Morganella/ or Morganella.mp. | 4113 |
| 45 | multidrug resistant tuberculosis/ or extensively drug resistant tuberculosis/ or Tuberculosis.mp. or drug resistant tuberculosis/ or tuberculosis/ | 263618 |
| 46 | TB.mp. | 85566 |
| 47 | Yersinia/ or Yersinia*.mp. | 20035 |
| 48 | Gram negative bacterium/ or Gram negative infection/ or Gram negative.mp. | 158332 |
| 49 | ciprofloxacin/ or ofloxacin/ or Fluoroquinolon*.mp. or fluoroquinolone resistance/ | 143965 |
| 50 | Cephalosporin*.mp. or cephalosporin/ or cephalosporin resistance/ | 71439 |
| 51 | carbapenem/ or Carbapenem*.mp. or carbapenem resistance/ | 39854 |
| 52 | Penicillin.mp. or penicillin derivative/ | 152271 |
| 53 | 32 or 33 or 34 or 35 or 36 or 37 or 38 or 39 or 40 or 41 or 42 or 43 or 44 or 45 or 46 or 47 or 48 or 49 or 50 or 51 or 52 | 2495751 |
| 54 | European Union.mp. or European Union/ | 42429 |
| 55 | EU.mp. | 51251 |
| 56 | European Union/ or Europe/ or European Economic Area.mp. | 184547 |
| 57 | European Union/ or EEA.mp. | 32538 |
| 58 | Austria.mp. or Austria/ | 40338 |
| 59 | Belgium.mp. or Belgium/ | 58328 |
| 60 | Britain.mp. or Great Britain/ | 28286 |
| 61 | Czech Republic.mp. or Czech Republic/ | 20752 |
| 62 | Denmark.mp. or Denmark/ | 86857 |
| 63 | England.mp. or United Kingdom/ or England/ | 492847 |
| 64 | Finland.mp. or Finland/ | 57048 |
| 65 | France.mp. or France/ | 237080 |
| 66 | Germany.mp. or Germany/ | 471033 |
| 67 | Greece/ or Greece.mp. | 36150 |
| 68 | Ireland/ or Ireland.mp. | 190353 |
| 69 | Iceland.mp. or Iceland/ | 8674 |
| 70 | Italy.mp. or Italy/ | 203692 |
| 71 | Lichtenstein.mp. | 1928 |
| 72 | Luxembourg.mp. or Luxembourg/ | 2311 |
| 73 | Netherlands/ or Netherlands.mp. | 158889 |
| 74 | Norway.mp. or Norway/ | 70684 |
| 75 | Portugal.mp. or Portugal/ | 29181 |
| 76 | Scotland.mp. or United Kingdom/ or Scotland/ | 415208 |
| 77 | Spain/ or Spain.mp. | 142967 |
| 78 | Sweden.mp. or Sweden/ | 135260 |
| 79 | Switzerland.mp. or Switzerland/ | 147441 |
| 80 | United Kingdom.mp. or United Kingdom/ | 549263 |
| 81 | UK.mp. or United Kingdom/ | 564522 |
| 82 | Wales/ or Wales.mp. | 34851 |
| 83 | 54 or 55 or 56 or 57 or 58 or 59 or 60 or 61 or 62 or 63 or 64 or 65 or 66 or 67 or 68 or 69 or 70 or 71 or 72 or 73 or 74 or 75 or 76 or 77 or 78 or 79 or 80 or 81 or 82 | 2703090 |
| 84 | 8 and 31 and 53 and 83 | 1092 |
| 85 | limit 84 to yr="2017 -Current" | 377 |

**Scopus**

| ( ( TITLE-ABS-KEY ( european  AND union )  OR  TITLE-ABS-KEY ( eu )  OR  TITLE-ABS-KEY ( european  AND economic  AND area )  OR  TITLE-ABS-KEY ( eea )  OR  TITLE-ABS-KEY ( european  AND free  AND trade  AND countr* )  OR  TITLE-ABS-KEY ( austria )  OR  TITLE-ABS-KEY ( belgium )  OR  TITLE-ABS-KEY ( britain )  OR  TITLE-ABS-KEY ( czech  AND republic )  OR  TITLE-ABS-KEY ( denmark )  OR  TITLE-ABS-KEY ( england )  OR  TITLE-ABS-KEY ( finland )  OR  TITLE-ABS-KEY ( france )  OR  TITLE-ABS-KEY ( germany )  OR  TITLE-ABS-KEY ( greece )  OR  TITLE-ABS-KEY ( ireland )  OR  TITLE-ABS-KEY ( iceland )  OR  TITLE-ABS-KEY ( italy )  OR  TITLE-ABS-KEY ( lichtenstein )  OR  TITLE-ABS-KEY ( luxembourg )  OR  TITLE-ABS-KEY ( netherlands )  OR  TITLE-ABS-KEY ( norway )  OR  TITLE-ABS-KEY ( portugal )  OR  TITLE-ABS-KEY ( scotland )  OR  TITLE-ABS-KEY ( spain )  OR  TITLE-ABS-KEY ( sweden )  OR  TITLE-ABS-KEY ( switzerland )  OR  TITLE-ABS-KEY ( united  AND kingdom )  OR  TITLE-ABS-KEY ( uk )  OR  TITLE-ABS-KEY ( wales ) ) )   AND  ( ( TITLE-ABS-KEY ( migrants* )  OR  TITLE-ABS-KEY ( migration )  OR  TITLE-ABS-KEY ( immigra* )  OR  TITLE-ABS-KEY ( refugee* )  OR  TITLE-ABS-KEY ( asylum )  OR  TITLE-ABS-KEY ( foreign  AND born )  OR  TITLE-ABS-KEY ( foreign-born ) ) )  AND  ( LIMIT-TO ( PUBYEAR ,  2023 )  OR  LIMIT-  TO ( PUBYEAR ,  2022 )  OR  LIMIT-TO ( PUBYEAR ,  2021 )  OR  LIMIT-TO ( PUBYEAR ,  2020 )  OR  LIMIT-TO ( PUBYEAR ,  2019 )  OR  LIMIT-TO ( PUBYEAR ,  2018 )  OR  LIMIT-TO ( PUBYEAR ,  2017 ) ) View less | AND | ( ( TITLE-ABS-KEY ( bacteri* )  OR  TITLE-ABS-KEY ( escherichia )  OR  TITLE-ABS-KEY ( e  AND coli )  OR  TITLE-ABS-KEY ( klebsiella* )  OR  TITLE-ABS-KEY ( staphylococcus )  OR  TITLE-ABS-KEY ( streptococcus )  OR  TITLE-ABS-KEY ( salmonell* )  OR  TITLE-ABS-KEY ( shigell* )  OR  TITLE-ABS-KEY ( gonorrhoeae )  OR  TITLE-ABS-KEY ( gonorrhea )  OR  TITLE-ABS-KEY ( proteus )  OR  TITLE-ABS-KEY ( enterobacter* )  OR  TITLE-ABS-KEY ( morganella )  OR  TITLE-ABS-KEY ( tuberculosis )  OR  TITLE-ABS-KEY ( tb )  OR  TITLE-ABS-KEY ( yersinia* )  OR  TITLE-ABS-KEY ( gram  AND negative )  OR  TITLE-ABS-KEY ( gram  AND positive )  OR  TITLE-ABS-KEY ( fluoroquinolon* )  OR  TITLE-ABS-KEY ( cephalosporin* )  OR  TITLE-ABS-KEY ( carbapenem* )  OR  TITLE-ABS-KEY ( penicillin ) ) )  AND  ( ( TITLE-ABS-KEY ( microbial  AND drug  AND resistan* )  OR  TITLE-ABS-KEY ( bacterial  AND resistan* )  OR  TITLE-ABS-KEY ( antibiotic  AND resistan* )  OR  TITLE-ABS-KEY ( antimicrobial  AND resistan* )  OR  TITLE-ABS-KEY ( xdr )  OR  TITLE-ABS-KEY ( mdr )  OR  TITLE-ABS-KEY ( multi-drug  AND resistant* )  OR  TITLE-ABS-KEY ( extensively  AND drug-resistan* )  OR  TITLE-ABS-KEY ( extensively  AND drug  AND resistant* )  OR  TITLE-ABS-KEY ( drug  AND resistan* )  OR  TITLE-ABS-KEY ( drug-resistan* )  OR  TITLE-ABS-KEY ( antimicrobial  AND resistan* )  OR  TITLE-ABS-KEY ( resistan* )  OR  TITLE-ABS-KEY ( non-susceptib* )  OR  TITLE-ABS-KEY ( decreased  AND susceptibility )  OR  TITLE-ABS-KEY ( beta-lactamase* )  OR  TITLE-ABS-KEY ( extended  AND spectrum  AND beta-lactamase* )  OR  TITLE-ABS-KEY ( esbl )  OR  TITLE-ABS-KEY ( ctx-m )  OR  TITLE-ABS-KEY ( amp  AND c )  OR  TITLE-ABS-KEY ( metallo-beta-lactamase* )  OR  TITLE-ABS-KEY ( mbl )  OR  TITLE-ABS-KEY ( methicillin-resistant  AND staphylococcus  AND aureus )  OR  TITLE-ABS-KEY ( mrsa ) ) ) |
| --- | --- | --- |
| Limits applied: Publication years 2017 to 2023 | | |
| 212 | | |

**PubMed**

| **(migrant* OR migration OR immigra* OR refugee* OR asylum OR foreign born OR foreign-born) AND (microbial drug resistan* OR bacterial resistan* OR antibiotic resistan* OR antimicrobial resistan* OR XDR OR MDR OR multi-drug resistan* OR extensively drug-resistan* OR extensively drug resistan* OR drug resistan* OR drug-resistan* OR antimicrobial resistan* OR resistan* OR non-susceptib* OR decreased susceptibility OR beta-lactamase* OR extended- spectrum beta-lactamase* OR ESBL OR CTX-M OR AmpC OR metallo-beta-lactamase* OR MBL OR methicillin-resistant Staphylococcus aureus OR MRSA) AND (bacteri* Escherichia OR E coli OR Klebsiella* OR Staphylococcus OR Streptococcus OR Salmonell* OR Shigell* OR Gonorrhoeae OR gonorrhea OR Proteus OR Enterobacter* OR Morganella OR Tuberculosis OR TB OR Yersinia* OR Gram negative OR Fluoroquinolon* OR Cephalosporin* OR Carbapenem* OR Penicillin) AND (European Union OR EU OR European Economic Area OR EEA OR European Free Trade countr* OR Austria OR Belgium OR Britain OR Czech Republic OR Denmark OR England OR Finland OR France OR Germany OR Greece OR Ireland OR Iceland OR Italy OR Lichtenstein OR Luxembourg OR Netherlands OR Norway OR Portugal OR Scotland OR Spain OR Sweden OR Switzerland OR United Kingdom OR UK OR Wales)** Filters: **from 2017 - 2023** | 330 results |
| --- | --- |

**Appendix II. Critical appraisal**

Table 3. Critical appraisal of cross-sectional studies, using the JBI checklist**.**

| Citation | Q1 | Q2 | Q3 | Q4 | Q5 | Q6 | Q7 | Q8 | Score (/8) |
| --- | --- | --- | --- | --- | --- | --- | --- | --- | --- |
| Creutz et al., 2022 | Y | Y | Y | Y | Y | Y | Y | Y | 8 |
| Ehlkes et al., 2019 | Y | Y | Y | Y | Y | Y | Y | Y | 8 |
| Eiset et al., 2020 | Y | Y | Y | Y | Y | Y | Y | Y | 8 |
| Hertting et al., 2021 | Y | Y | Y | Y | Y | U | Y | Y | 7 |
| Kenfak et al., 2021 | Y | Y | Y | U | N | N | Y | Y | 5 |
| Mellou et al., 2021 | Y | Y | Y | Y | Y | U | Y | Y | 7 |
| Najem et al., 2022 | Y | Y | Y | Y | Y | Y | Y | Y | 8 |
| Nurjadi et al., 2019 | Y | Y | Y | Y | U | N | Y | Y | 6 |
| Stabler et al., 2021 | Y | Y | Y | Y | Y | Y | Y | Y | 8 |
| Van-Dulm et al., 2020 | Y | Y | Y | Y | U | U | U | Y | 5 |

**Keys: Y=Yes, N=NO, U=Unclear Q1.Were the criteria for inclusion in the sample clearly defined? Q2.Were the study subjects and the setting described in detail? Q3.Was the exposure measured in a valid and reliable way? Q4.Were objective, standard criteria used for measurement of the condition? Q5.Were confounding factors identified? Q6.Were strategies to deal with confounding factors stated? Q7.Were the outcomes measured in a valid and reliable way? Q8.Was appropriate statistical analysis used?**

Table 4. Critical appraisal of cohort studies, using the JBI checklist.

| Citation | Q1 | Q2 | Q3 | Q4 | Q5 | Q6 | Q7 | Q8 | Q9 | Q10 | Q11 | Score (/11) |
| --- | --- | --- | --- | --- | --- | --- | --- | --- | --- | --- | --- | --- |
| Aro et al., 2018 | Y | Y | Y | Y | Y | Y | Y | U | U | U | Y | 8 |
| Costa et al., 2018 | Y | Y | Y | Y | Y | Y | Y | Y | U | U | Y | 9 |
| Garriga et al., 2021 | Y | Y | Y | Y | Y | Y | Y | Y | U | U | Y | 9 |
| Kossow et al., 2018 | Y | Y | Y | Y | U | Y | Y | Y | Y | N | Y | 9 |
| Lemoine et al., 2022 | Y | Y | Y | Y | Y | Y | Y | U | U | U | Y | 8 |
| Fiorini et al., 2022 | Y | Y | Y | Y | Y | U | Y | U | U | U | Y | 7 |
| Ravensbergen et al., 2019 | Y | Y | Y | Y | Y | Y | Y | Y | Y | Y | Y | 11 |
| Reinheimer et al., 2019 | Y | Y | Y | Y | Y | Y | Y | Y | Y | U | Y | 9 |
| Rovirola et al., 2020 | Y | U | Y | Y | Y | Y | Y | Y | Y | N | N | 8 |
| Saracino et al., 2020 | Y | U | Y | Y | Y | Y | Y | U | U | U | Y | 7 |
| Sloth et al., 2019 | Y | Y | Y | Y | Y | Y | Y | Y | Y | Y | Y | 11 |

**Keys: Y=Yes, N=NO, U=Unclear NA=Not applicable Q1.Were the two groups similar and recruited from the same population? Q2.Were the exposures measured similarly to assign people to both exposed and unexposed groups Q3.Was the exposure measured in a valid and reliable way? Q4 Were confounding factors identified? Q5. Were strategies to deal with confounding factors stated? Q6.Were groups free of the outcome at the start of study? Q7.Were the outcomes measured in a valid and reliable way? Q8. Was the follow up time reported and sufficient to be long enough for outcomes to occur?Q9.was follow up complete and if not were reasons to loss to follow up described and explored?Q10.were strategies to address incomplete follow up utilisedQ11.Was appropriate statistical analysis used?**

**
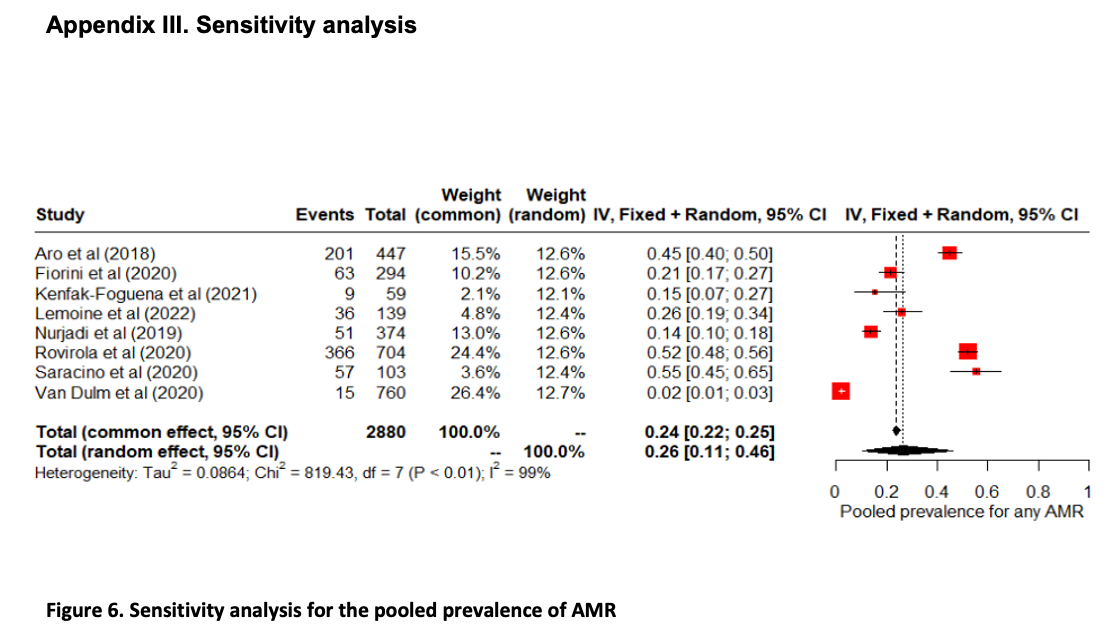
**

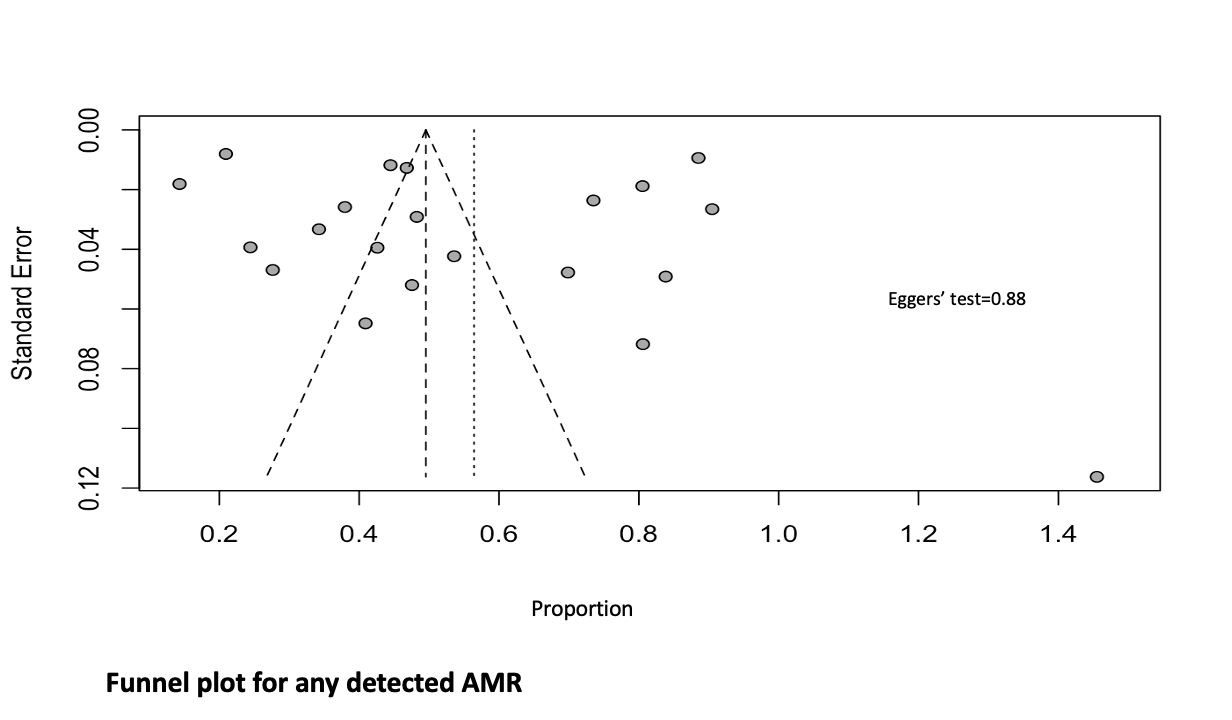


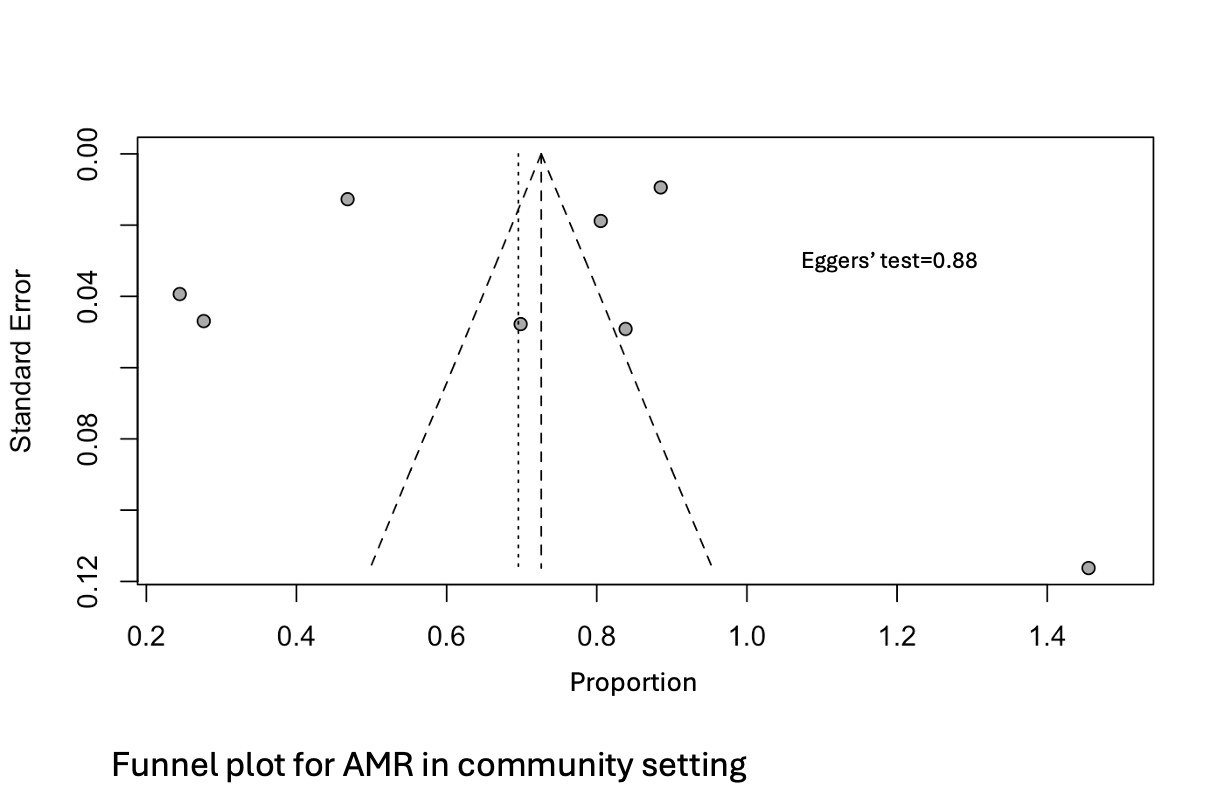


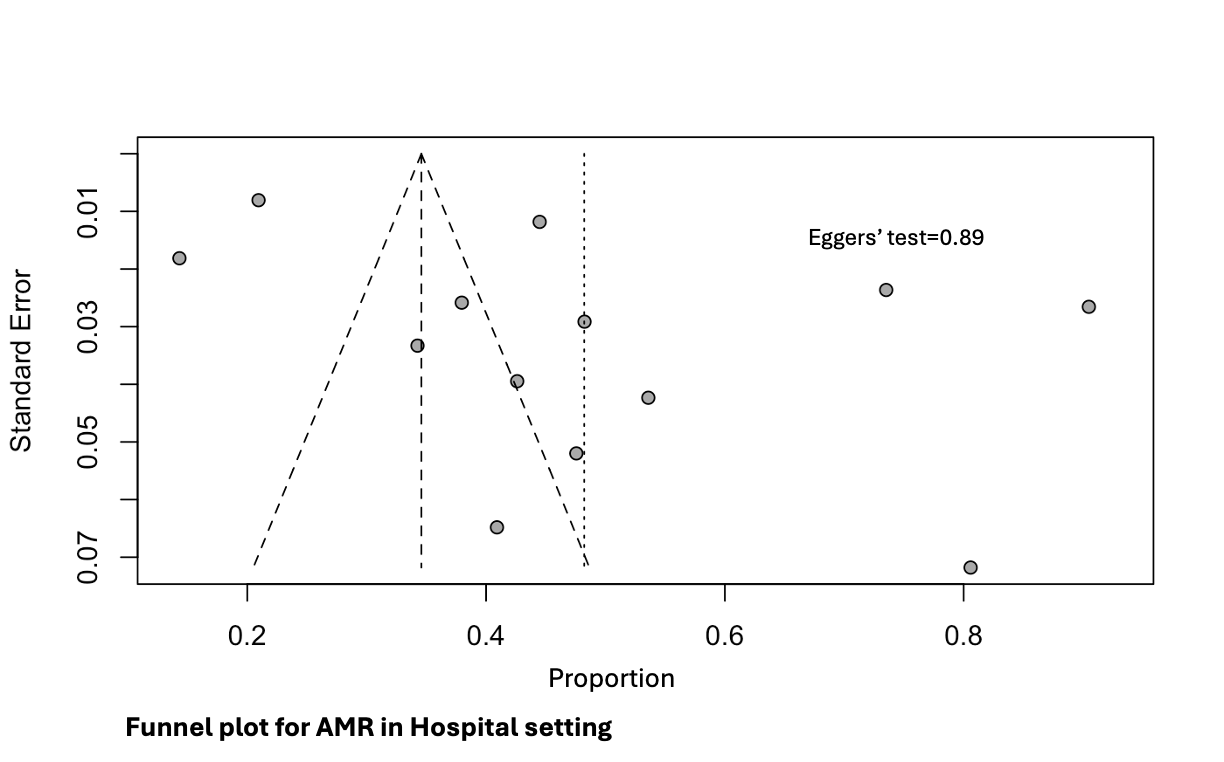


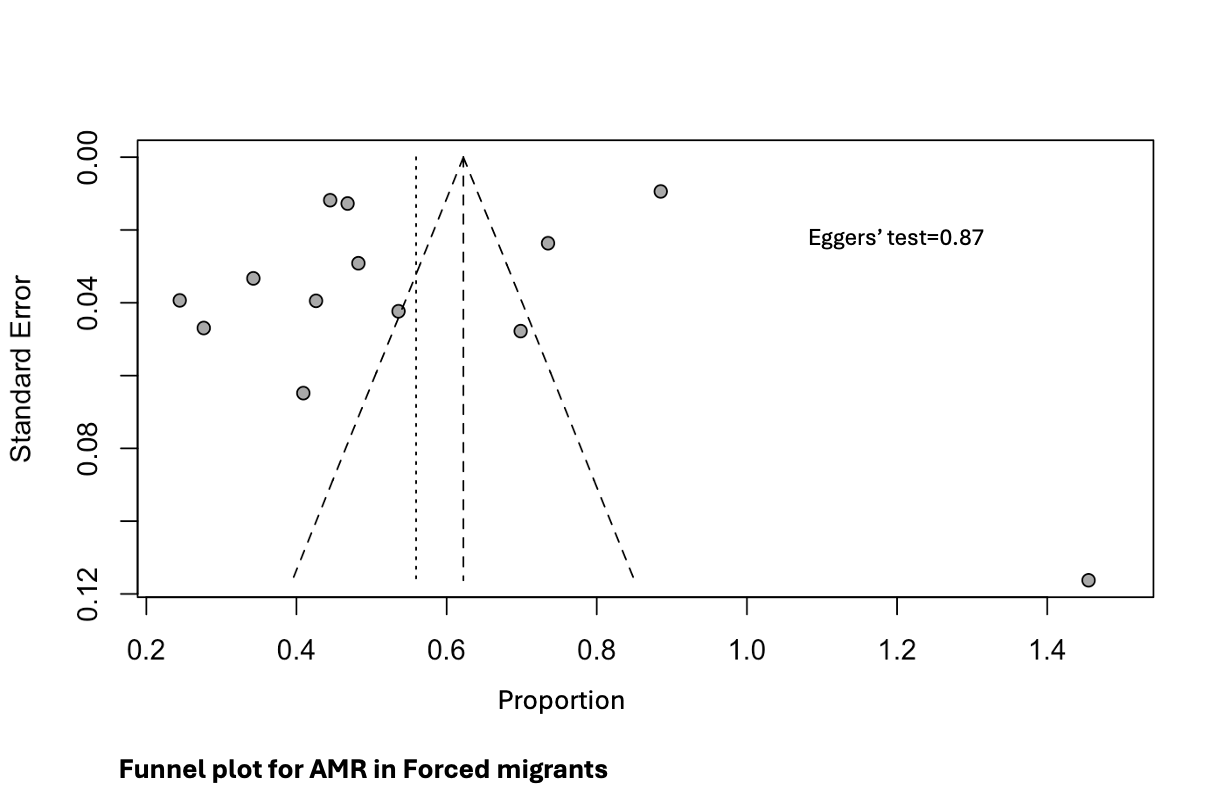


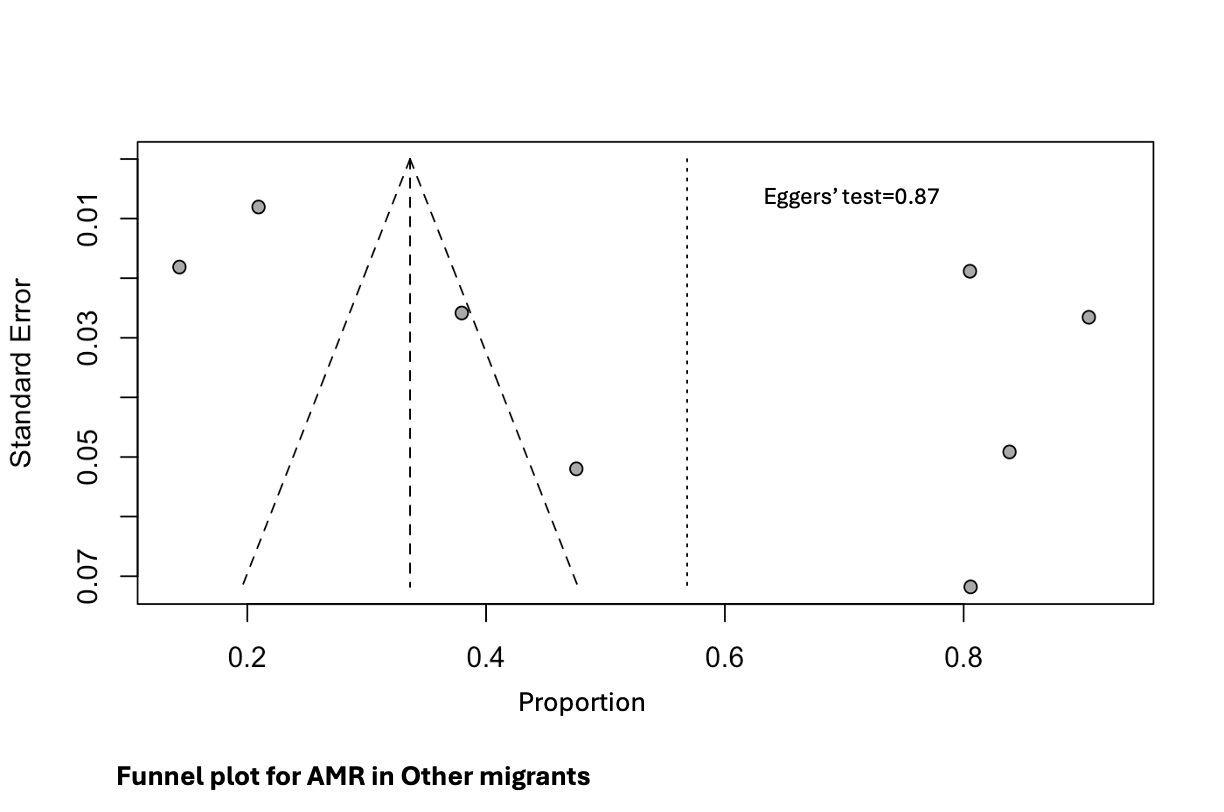

Supplement: Supplementary Tables and Figures [file mmc1.docx]
